# Supplementary material for: A Lipidomics Study Reveals Lipid Signatures Associated with Early Allograft Dysfunction in Living Donor Liver Transplantation
Source: J Clin Med. 2018 Dec 29;8(1):30. doi: 10.3390/jcm8010030 (PMC6352109; doi:10.3390/jcm8010030)
Supplement: Supplementary file 1 [file jcm-08-00030-s001.pdf]

Supplementary Materials

Table S1. Demographic details from the validation study population.

Validation Group	EAD [11] (n = 9)	Non-EAD [11] (n = 12)	p-Value
Height (m)	1.66 ± 0.06	1.58 ± 0.05	<0.01
Weight (kg)	69.89 ± 10.13	64.00 ± 10.19	0.20
BMI [1]	25.49 ± 3.43	25.63 ± 3.11	0.92
Age (years)	47.11 ± 7.77	58.18 ± 6.75	<0.01
Gender			<0.01
M/F	8/1	4/8	
Blood type			0.68
A/B/O/AB	2/3/3/1	3/4/5/0	
MELD [2]	23.78 ± 11.68	15.50 ± 9.57	0.09
HBV [3] (Yes/No)	5/4	5/7	0.55
HCV [4] (Yes/No)	2/7	6/6	0.21
Alcoholism (Yes/No)	3/6	2/10	0.40
HCC [5] (Yes/No)	3/6	7/5	0.28
ABO incompatibility (Yes/No)	1/8	2/10	0.73
CKD [6] (Yes/No)	3/6	1/11	0.16
Blood loss (mL)	2116.67 ± 1290.83	1733.33 ± 1184.24	0.49
Graft (g)	626.22 ± 120.84	614.17 ± 123.62	0.83
GRWR [7] (%)	0.92 ± 0.22	0.95 ± 0.24	0.34
Cold ischemia (min)	47.67 ± 22.61	46.67 ± 25.40	0.93
Warm ischemia (min)	52.33 ± 37.72	75.08 ± 75.60	0.42
PRBC [8]	10.00 ± 6.56	6.50 ± 5.98	0.22
FFP [9]	18.67 ± 11.18	8.00 ± 7.29	0.02
Platelet	6.67 ± 8.72	6.17 ± 7.98	0.89
In hospital mortality	0.29 ± 0.49	0.00 ± 0.00	N/A
ICU [10] stay	20.00 ± 12.81	13.25 ± 8.96	0.17
Hospital stay	50.38 ± 18.47	40.30 ± 28.10	0.40

[1] body mass index; [2] model for end-stage liver disease; [3] hepatitis B virus; [4] hepatitis C virus; [5] hepatocellular carcinoma; [6] chronic kidney disease; [7] graft recipient weight ratio; [8] packed red blood cell; [9] fresh frozen plasma; [10] intensive care unit; [11] early allograft dysfunction.

Table S2. Biochemical details from the validation study population.

Validation	EAD [3] (n = 9)	Non-EAD [3] (n = 12)	p-Value
Histidine (μM)	114.1 ± 32.5	83.3 ± 14.1	0.0234
Asparagine (μM)	76.9 ± 29.8	64.3 ± 17.2	0.2380
Taurine (μM)	48.4 ± 20.7	40.1 ± 10.3	0.2987
Serine (μM)	111.0 ± 45.0	126.0 ± 31.2	0.3782
Glutamine (μM)	638.3 ± 172.2	569.5 ± 180.9	0.3898
Arginine (μM)	101.2 ± 48.1	89.9 ± 36.2	0.5478
Glycine (μM)	274.2 ± 105.6	314.4 ± 131.5	0.4608
Citrulline (μM)	39.3 ± 13.3	82.4 ± 163.4	0.3826
Glutamate (μM)	46.0 ± 29.9	59.0 ± 19.8	0.2571
Threonine (μM)	163.9 ± 52.8	183.0 ± 61.4	0.4653
Alanine (μM)	277.0 ± 142.7	268.2 ± 83.2	0.8609
Proline (μM)	189.4 ± 60.3	189.1 ± 89.4	0.9942

Ornithine (μM)	54.8 ± 30.9	49.0 ± 17.4	0.5919
Cystine (μM)	60.8 ± 33.0	70.6 ± 20.3	0.4083
Lysine (μM)	242.4 ± 91.8	230.1 ± 82.7	0.7519
Tyrosine (μM)	103.4 ± 44.8	71.8 ± 22.3	0.0772
Methionine (μM)	118.7 ± 228.9	36.6 ± 18.4	0.3138
Valine (μM)	261.6 ± 105.0	203.2 ± 74.2	0.1515
Isoleucine (μM)	75.4 ± 37.8	67.2 ± 17.4	0.5587
Leucine (μM)	117.5 ± 46.1	98.6 ± 28.9	0.2626
Phenylalanine (μM)	113.1 ± 45.0	72.4 ± 23.8	0.0146
Tryptophan (μM)	63.2 ± 32.0	66.5 ± 21.1	0.7764
Essential AA (μM)	1310.1 ± 496.7	1046.1 ± 286.4	0.1404
BCAA [1] (μM)	454.4 ± 184.7	369.0 ± 111.3	0.2025
AAA [2] (μM)	279.7 ± 111.5	210.7 ± 51.4	0.1137
Fischer's ratio	1.8 ± 0.9	1.8 ± 0.4	0.9762

[1] aromatic amino acids; [2] branched chain amino acids; [3] early allograft dysfunction.

Supplementary Methods

NMR Analysis of the Plasma

Before the NMR analysis, the frozen plasma samples were thawed on ice. Next, 350 μL of the plasma sample was mixed with 350 μL of a plasma buffer solution (75 mM Na_2HPO_4, 0.08% TSP, 2 mM NaN_3, 20% D_2O), and the mixed sample was centrifuged at 12,000 *g* at 277 K for 5 min. Finally, 600 μL of the supernatant was transferred to 5 mm NMR tubes for analysis.

^{1}H NMR spectra were acquired on a Bruker Avance III HD 600 MHz NMR spectrometer at 310 K using a 5 mm inverse triple resonance CryoProbe (^{1}H/^{13}C/^{15}N) (Bruker Biospin GmbH, Rheinstetten, Germany). The spectra were acquired by Carr-Purcell-Meiboom-Gill (CPMG) spin-echo pulse sequence. The total T_2 relaxation time of 80 ms was used to attenuate broad signals from proteins, and the spectrum was collected with a spectral width of 12019.23 Hz. Free induction decay (FID) was acquired into 72k data points and the acquisitions were accumulated 32 times. FIDs were weighted by an exponential function with a 0.3 Hz line broadening factor prior to Fourier transformation. All NMR spectra were phased and baseline-corrected using Topspin software (version 3.2.2; Bruker Biospin GmbH, Rheinstetten, Germany), then referenced to the doublet of 1H-α-glucose at 5.23 ppm.

Each ^{1}H NMR spectrum from plasma was segmented into equal widths (0.01 ppm), corresponding to regions 9.5-0.5 ppm, and the spectral data were normalized to the reference by AMIX (version 3.9.14; Bruker Biospin GmbH, Rheinstetten, Germany). The resulting data sets were analysed by SIMCA-P+ (version 13.0; Umetrics, Umea, Sweden), and all data were UV-scaled for multivariate statistical analysis. Principal components analysis (PCA), an unsupervised pattern recognition method, was performed to examine the intrinsic variation in the data set. Orthogonal partial least squares-discriminant analysis (OPLS-DA) was used to maximize covariance between the measured data (peak area from the NMR spectra) and the response variable (predictive classifications). Resonant frequencies of each metabolite were

referred from library of in-house, Chenomx NMR Sutie 7.1 (Chenomx, Edomonton, Canada) (17), or HMDB (http://www.hmdb.ca/).

LCMS-Based Lipidomic

To 10-µl plasma, 490 µl isopropanol (IPA) (precooling at −20 °C) was added. The mixture was vortexed for 60 s, let stand on ice 30 min, and centrifuged at 12,000 rpm at 4 °C for 30 min. The supernatant was collected in a separate glass tube, diluted with IPA/acetonitrile (ACN)/H_2O(2/1/1) and centrifuged at 12,000 rpm for 30 min. The clear supernatant was collected for LC-MS analysis.

The liquid chromatographic separation was achieved on a 100 mm × 2.1 mm Acquity 1.7-µm C18 column (Waters Corp., Milford, MA, USA) using an ACQUITY TM UPLC system (Waters Corp., Milford, MA, USA). The column was maintained at 60 °C, with a flow rate of 0.45 mL/min. The samples were eluted from the LC column using a linear gradient: 0–10 min: 40–99% B; 10.1–12 min: 40% B for re-equilibration. The mobile phases were ACN/H_2O (40:60) with 10 mM ammonium formate (solvent A) and IPA/ACN (90:10) with 10 mM ammonium formate (solvent B).

The eluent was introduced into a TOF MS system (SYNAPT G1 high-definition mass spectrometer, Waters Corp., Milford, MA, USA) operated in an ESI-positive ion mode. The conditions were as follows: the desolvation gas was set to 800 l/h at a temperature of 400 °C, the cone gas was set to 25 L/h, and the source temperature was set at 100 °C. The capillary voltage and the sample cone voltage were set to 2700 V and 35 V, respectively. The MCP detector voltage was set to 1750 V. The data acquisition rate was set at 0.2 s with a 0.02 s interscan delay. The data were collected in centroid mode from 20 to 990 m/z. For accurate mass acquisition, a lock-mass of leucine enkephalin at a concentration of 60 ng/mL and a flow rate of 6 L/min was used (an $[M+H]^+$ ion at 556.2771 Da in ESI-positive mode).

Raw mass spectrometric data were processed using Progenesis QI software (Waters Corp., Milford, MA, USA). The intensity of each mass ion was normalized with respect to the total ion count to generate a data matrix that included the retention time, m/z value, and the normalized peak area. The multivariate data matrix was analyzed using the SIMCA-P software. OPLS-DA models were carried out prior to the application of Pareto scaling. SIMCA-P had been used for the multivariate data analysis and representation.

Exact molecular mass data that showed significant differences between two groups were then submitted for database searching, either in-house or using the online HMDB (http://www.hmdb.ca/) and KEGG (http://www.genome.jp/kegg/) databases. For identification of targeted metabolites, MS/MS spectra were acquired and compared with in-house library, HMDB or METLIN (http://metlin.scripps.edu/) database.

Ultra-Performance Liquid Chromatography (UPLC)-Based Amino Acid Measurement

The plasma samples were collected and stored at −80 °C until assayed. The plasma samples (100 µL) were precipitated by adding an equal volume (100 µL) of 10% sulfosalicylic

acid containing an internal standard (norvaline 200 μM) [19]. After protein precipitation, the samples were vortexed and centrifuged at 12,000 *g* for 10 min at room temp. After the samples were centrifuged, 20 μL of the supernatant was mixed with 60 μL working buffer (borate buffer, pH 8.8). The derivatization was initiated by adding 20 μL of 10 mM AQC in acetonitrile. After 10 min incubation, the reactant was mixed with an equal volume of Eluent A (20 mM ammonium formate/0.6% Formic acid/1% acetonitrile) and analyzed using the ACQUITY UPLC System. The AQC derivatization reagent was obtained from the Waters Corporation (Milford, MA, USA). An aqueous 22 amino acid standard mixture was prepared at different concentrations (0, 25, 50, 100, 250, 500 μM) for each amino acid and analyzed by the same procedure.

The Waters ACQUITY UPLC® System consisted of a Binary Solvent Manager (BSM), a Sample Manager fitted with a 10-μL loop, and a Tunable UV (TUV) detector. The system was controlled, and the data was collected using Empower™ 2 software. The separations were performed on a 2.1 × 100 mm ACQUITY BEH C18 column at a flow rate of 0.70 mL/min. The column temperature was set at 60 °C, and the injection volume for all samples and standards was 2.0 μL. Water/acetonitrile (95:5) was used as the weak needle wash solvent, and water/acetonitrile (5:95) was used as the strong needle wash solvent. The analytes were eluted from the column using a linear gradient: 0–0.54 min: 0.1% B; 0.54–5.74 min: 0.1–9.1% B; 5.74–7.74 min: 9.1–21.2% B; 7.74–8.0 min: 21.2–59.6% B; 8.0–8.05 min: 59.6–90% B; 8.05–8.64 min: 90% B; 8.64–8.73 min: 0.1% B; 8.73–9.5 min for re-equilibration. The mobile phase was 20 mM ammonium formate/0.6% formic acid/1% acetonitrile in water (Eluent A) and in acetonitrile (Eluent B). The detection was set at 260 nm using a sampling rate of 20 points per second.
